# A safety catch for ornithine decarboxylase degradation

**DOI:** 10.15698/mic2015.06.210

**Published:** 2015-05-27

**Authors:** Christof Taxis

**Affiliations:** 1 Department of Biology/Genetics, Philipps-University Marburg, Germany.

**Keywords:** degron, feedback inhibition, ornithine decarboxylase, polyamines, proteasome, protein degradation, ubiquitin-independent degradation

**Figure 1:**
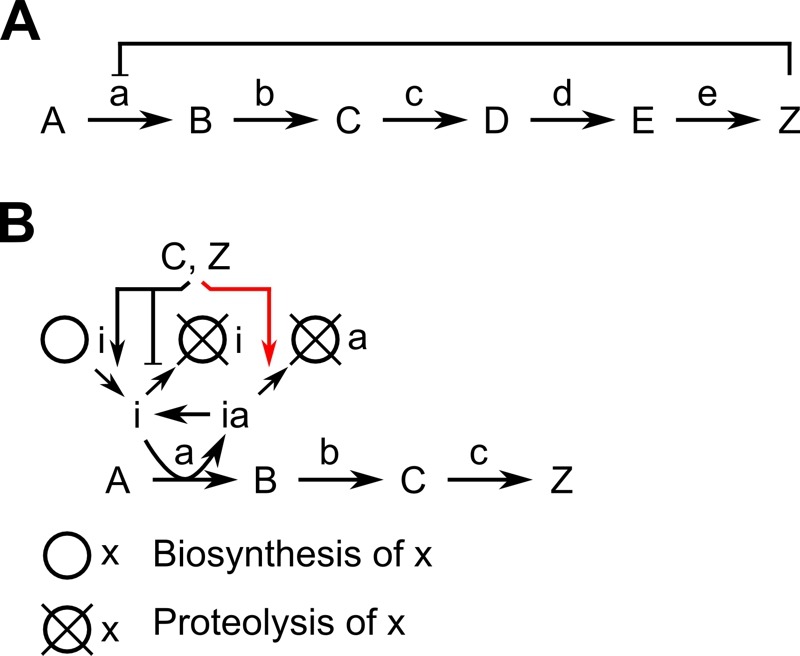
FIGURE 1: Feedback inhibition of enzymes. **(A)** Classical regulation: An end-product (Z) of a biosynthesis pathway inhibits activity of an enzyme (a) catalyzing the first irreversible step (commitment step). **(B)** The polyamines spermidine (C) and spermine (Z) regulate the activity of ornithine decarboxylase (ODC; a) by inducing the biosynthesis of the ODC-inhibitor antizyme (i) and preventing its degradation. Binding of antizyme to ODC (symbolized by ia) inhibits ODC activity and induces its degradation; a novel finding is that polyamines directly promote antizyme-mediated degradation of ODC (highlighted by red color).

Feedback inhibition is a common mechanism to adjust the activity of an enzyme in accordance with the abundance of a product. The enzyme catalyzing the initial, committing step of a biosynthesis cascade is subject to negative feedback by the end-product. This kind of regulation is frequent in all cell types to regulate biosynthesis of numerous metabolites; a classical textbook example is the serine biosynthesis pathway 1. The first irreversible reaction (A->B) is regulated by the end-product (Z) of the biosynthesis pathway (Figure 1A). In *E. coli*, serine inhibits 3-phosphoglycerate dehydrogenase activity by binding to a regulatory site within the enzyme. Thus, the enzyme is inactive whenever serine is in excess. Negative feedback can be implemented also in a different way: the end-product influences abundance and activity of a protein-based inhibitor, which regulates the enzyme catalyzing the initial step, to control the flux through the biosynthesis pathway. An example for this kind of feedback inhibition in eukaryotic cells is the regulation of polyamine-levels by feedback inhibition of ornithine decarboxylase (ODC) (Figure 1B), which is rate-limiting for the synthesis of the aliphatic polyamines putrescine, spermidine and spermine 2,3.

These polyamines are aliphatic polycations that influence mRNA translation, ribosome biogenesis and bind and stabilize RNA as well as DNA. Thus, numerous processes are regulated by polyamines directly or indirectly. They are essential for cell proliferation, yet, their over-abundance is cytotoxic and involvement in cell death has been observed in higher eukaryotes. Therefore, polyamine uptake and biosynthesis has to be efficiently controlled to balance polyamine levels, which requires tight feedback inhibition of ODC to maintain polyamine levels in a nontoxic concentration range. During development of most human cancers a deregulation of polyamine biosynthesis takes place. Due to the importance of polyamines for cell proliferation, ODC and polyamine biosynthesis have been chosen as anti-cancer targets 4.

Regulation of ODC activity is based on the protein-inhibitor ODC antizyme; members of this protein family have been identified in yeast, protists and mammals 5,6. Biosynthesis and proteolysis of antizyme is under feedback inhibition by polyamines. The inhibitor binds ODC, converts the enzymatically active dimer into an inactive heterodimer and induces degradation of ODC 4. Feedback inhibition of polyamine biosynthesis is accomplished by increasing the abundance of the inhibitor antizyme and decreasing the abundance of ODC, the enzyme catalyzing the rate-limiting step (Figure 1B).

By concept, polyamine biosynthesis levels are controlled in a simple way, but the details of how regulation is achieved are quite interesting. A conserved mechanism controls translation of antizyme mRNA in yeast, protists and mammals. The antizyme-encoding gene is divided in two parts with the second open reading frame shifted by one nucleotide with respect to the first one 7. Thus, translation of the whole antizyme requires a +1 ribosomal frameshift event after the initial part of the nascent polypeptide chain is decoded 6,8. It was shown in yeast that the frameshift is initiated upon association of polyamines with the amino-terminal part of the antizyme polypeptide 9. Thus, only if the polyamine concentration is high in a cell, the antizyme transcript is translated completely 10.

After folding, antizyme will bind to ODC; thereby the active ODC-dimer is transformed into inactive heterodimers consisting of antizyme and ODC. This leads to exposure of a degradation-inducing sequence (degron) resulting in proteolysis of ODC. The breakdown of ODC by proteasomes is somewhat unconventional; its proteolysis is independent of ubiquitin and does not rely on modification with a polyubiquitin chain as degradation signal 11,12. The degradation mechanism itself is conserved from yeast to man, yet the actual degrons differ considerably. Yeast ODC contains an amino-terminal degron called ornithine degradation sequence (ODS), which consists of an unstructured region of roughly 50 amino acids followed by sequences folded into an α-helix. Both features are necessary to act as ubiquitin-independent degron 11. Mammalian ODC was the first substrate of the proteasome that was identified to be degraded by an ubiquitin-independent degradation mechanism 13. The degron is located at the carboxy-terminal end and consists of 37 amino acids. Essential requirements are a cysteine-alanine motif and the absence of secondary structure elements within this region 14. The murine ODC degron has been proven to be a versatile tool for synthetic approaches that influence protein stability due to the transferability and the tolerance towards changes in its sequence 15,16,17,18. Even though the degrons in yeast and mammalian ODC are located at different ends of ODC, they share key features, including exposure upon antizyme binding and tolerance towards exchanges in their sequence 11,14.

**Figure 2:**
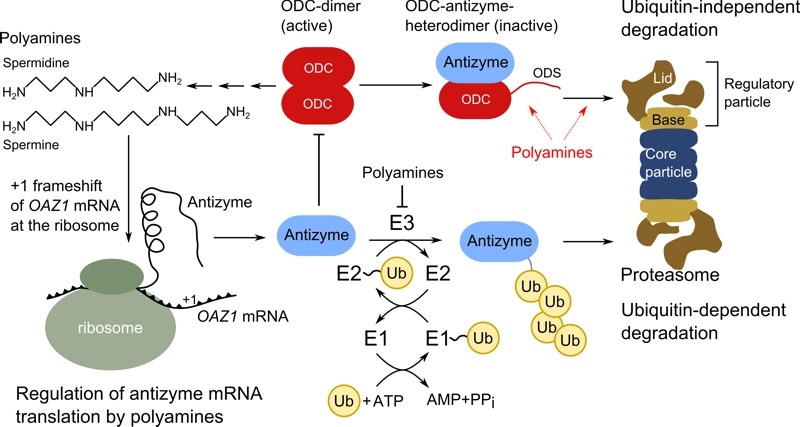
FIGURE 2: Regulation of ornithine decarboxylase activity by polyamines in yeast. Ornithine decarboxylase (ODC) is an enzyme that catalyzes the rate-limiting commitment step in polyamine biosynthesis. It is a dimer in its active form and is inhibited by antizyme, which binds to ODC in a one-to-one complex as heterodimer. The yeast gene encoding antizyme is called *OAZ1*, which produces an mRNA with two ORFs. To produce the full protein, a +1 frameshift during antizyme mRNA translation is necessary at the ribosome, which is induced by the polyamines spermidine or spermine. Antizyme does not have to be present in large amounts to inhibit ODC activity, as binding of ODC by antizyme leads to presentation of the ODC degradation sequence (ODS), which induces proteasomal degradation. This step is directly promoted by polyamines. Polyamines could increase ODS presentation, enhance binding of antizyme to proteasomes, or both (dashed red arrows). Antizyme itself escapes destruction by the proteasome, thus even low levels of antizyme catalyze degradation of ODC. Antizyme stability is regulated by polyamines as well; the presence of polyamines reduces antizyme proteolysis by an ubiquitin-dependent proteasomal degradation process. The latter depends on the ubiquitin-activating enzyme (E1) Uba1, the ubiquitin-conjugating enzymes Ubc4 and Ubc5 (E2), and an unidentified ubiquitin-protein ligase (E3). Overall, polyamines influence ODC activity threefold; they enhance ODC proteolysis, increase antizyme levels by promoting its biosynthesis and inhibit its destruction, which provides a negative feedback loop for ODC regulation by polyamines.

Beenukumar *et al.* report in this issue of *Microbial Cell* a novel tweak in feedback inhibition of ODC by polyamines in yeast. Their investigations demonstrate that polyamines directly promote the degradation of ODC and not only indirectly by regulating abundance of the ODC inhibitor antizyme, as has been assumed previously 19. Thus, efficient break-down of the rate-limiting enzyme of polyamine biosynthesis occurs only if polyamines are present in the cell (Figure 2). This provides a safety catch on degradation of the enzyme and inhibits premature degradation of ODC, which could occur upon erroneous synthesis of antizyme. Importantly, this is the first example of a small natural compound having a stimulatory effect on proteasomal proteolysis of a specific substrate 19.

To uncover this novel finding, the Dohmen lab had to use elaborate *in vivo* and *in vitro *experiments. For each test they got the same result: antizyme alone triggers degradation of ODC not as efficiently as antizyme plus a polyamine like spermidine or spermine. *In vitro,* a maximized ODC degradation rate was observed in experiments combining the polyamine spermine with a mixture of purified proteasomes, ODC, and antizyme. The crucial point for the *in vivo* experiments was to uncouple antizyme-levels from the influence of polyamines, which demonstrated convincingly that polyamines promote ODC degradation. Here, spermidine showed enhanced capabilities to promote ODC degradation compared to spermine. The difference in effectiveness of spermine and spermidine *in vitro* and *in vivo* still awaits a clarifying explanation. However, polyamines are not simply beneficial for the activity of proteasomes, as degradation of unrelated proteasomal substrates were not influenced by changes in polyamine levels 19.

The finding of the Dohmen group that ODC degradation is directly enhanced by polyamines reveals an additional layer of regulation. This might be an important tweak that is necessary to fine-tune ODC activity and thereby intracellular levels of polyamines. A remaining question is whether a similar regimen exists in higher eukaryotes. So far, regulation of ODC activity was found to be astonishingly conserved from yeast to man. Overall, antizyme regulates ODC activity in a twofold manner: it leads to the formation of enzymatically inactive heterodimers and induces the degradation of ODC. Yet, polyamines influence ODC activity in three ways, by stimulating antizyme biosynthesis, inhibiting its degradation and promoting ODC degradation (Figure 2). Uncovering the regulatory details of polyamine biosynthesis is not only rewarding due to the uncommon mechanistic details hidden in this pathway, but is also of importance due to the numerous cellular functions influenced by polyamines.
